# Integrative transcriptomics nominates a 1R-MYB candidate associated with epidermal wax-related traits and osmotic-stress responses in Kentucky bluegrass

**DOI:** 10.3389/fpls.2026.1817480

**Published:** 2026-05-20

**Authors:** Xue You, Yifeng Jin, Xiaoxue Liang, Qi Wang, Haoran Yu, Yuwen Wang, Yang Chen, Miao He

**Affiliations:** 1 College of Life Science and Agriculture Forestry, Qiqihar University, Qiqihar, China; 2 College of Chemistry, Chemical Engineering and Resource Utilization, Northeast Forestry University, Harbin, China; 3 College of Landscape Architecture, Northeast Forestry University, Harbin, China

**Keywords:** 1R-MYB, cuticular wax, drought tolerance, Phytohormone signaling, Poa pratensis L., root architecture, ROS, transcriptome

## Abstract

Cuticular wax-associated epidermal traits may contribute to water-loss regulation, yet their regulatory basis in Kentucky bluegrass (*Poa pratensis* L.) remains poorly defined. Here, we combined comparative phenotyping and *de novo* transcriptomics across three cultivars exhibiting contrasting wax-associated epidermal structures. Scanning electron microscopy revealed significant cultivar differences in wax crystal density (quantified as crystals per 100 μm^2^), and a pot water-withholding assay showed cultivar-dependent injury phenotypes accompanied by differential ROS staining, antioxidant enzyme activities, relative water content, and lipid peroxidation levels. *De novo* transcriptome assembly (BUSCO completeness 76.8%) of pooled reads from nine libraries identified 228310 unigenes, among which pairwise comparisons revealed 7257–29713 differentially expressed genes with recurrent enrichment of hormone signal transduction pathways. A multi-criteria filtering strategy highlighted a 1R-MYB gene (*PpMYBS3*) displaying the strongest expression concordance with the cultivar wax-density gradient and progressive stress inducibility by qRT-PCR. Stable overexpression of *PpMYBS3* in two independent transgenic lines increased wax-associated epidermal structures, upregulated canonical wax biosynthesis genes (*CER1*, *KCS6*, *ABCG11*), reduced stomatal aperture area, enhanced root architecture traits, and mitigated ROS accumulation under PEG-induced osmotic stress. These results support *PpMYBS3* as a candidate regulator sufficient to promote epidermal and root traits linked to stress adaptation, while emphasizing that direct quantification of wax composition (e.g., GC-MS wax profiling) and cuticular water-loss assays will be required to establish mechanistic causality.

## 1 Introduction

Lawns provide essential ecological services including soil erosion prevention, carbon sequestration, and oxygen production, representing critical components of urban green infrastructure (Xu et al., 2011; Guillard and Inguagiato, 2017). Kentucky bluegrass (*Poa pratensis* L.), valued for its fine texture and excellent turf quality, is extensively utilized in parks, golf courses, and residential landscapes worldwide. However, this species exhibits notable susceptibility to drought stress, a challenge that has intensified under climate change scenarios (Bushman et al., 2013; Blum, 2017). Under water deficit conditions, Kentucky bluegrass enters summer dormancy characterized by growth cessation and quality deterioration, substantially increasing maintenance costs and reducing aesthetic value (Bushman et al., 2021). Therefore, elucidating the molecular mechanisms underlying drought tolerance and developing stress-resistant varieties constitutes a critical priority for sustainable turfgrass management.

Plants have evolved multiple adaptive mechanisms to cope with water deficit, among which the cuticular wax layer serves as the primary barrier against non-stomatal water loss (Patwari et al., 2019). The plant cuticle, composed of cutin polymer and cuticular waxes, covers the aerial surfaces of terrestrial plants and plays indispensable roles in limiting transpiration, reflecting ultraviolet radiation, and providing defense against biotic stressors (Yang et al., 2011). Studies in apple (*Malus domestica*) have demonstrated that enhanced expression of wax biosynthesis genes significantly increases epidermal wax accumulation and improves drought resistance (Zhang et al., 2019). Similarly, dysfunction of wax synthesis genes in rice (*Oryza sativa*) reduces cuticular wax density and diminishes drought tolerance (Jian et al., 2022). In *Poa annua* L., a strong negative correlation has been established between epidermal wax coverage and electrolyte leakage under stress conditions, indicating the protective role of wax in stress mitigation (Chalise and Merewitz, 2024). However, the genetic regulation of cuticular wax biosynthesis in Kentucky bluegrass remains poorly characterized.

Phytohormone signaling networks integrally regulate plant responses to drought stress. Abscisic acid (ABA) serves as the central hormonal regulator of drought responses, modulating stomatal closure through the PYR/PYL-PP2C-SnRK2 signaling cascade (Vlad et al., 2009; Nishimura et al., 2010). The 9-cis-epoxycarotenoid dioxygenase 3 (*NCED3*) gene, encoding a rate-limiting enzyme in ABA biosynthesis, is rapidly induced by drought stress in *Arabidopsis thaliana* (Takahashi et al., 2018). Auxin (indole-3-acetic acid, IAA) also contributes to drought adaptation by modulating root architecture and serving as a reactive oxygen species scavenger (Kaya et al., 2018; Riseh et al., 2021). The interplay between ABA and auxin signaling pathways constitutes a complex regulatory network that fine-tunes plant responses to water deficit; however, this crosstalk remains insufficiently understood in Kentucky bluegrass.

MYB transcription factors comprise one of the largest transcription factor families in plants, participating in diverse biological processes including development, metabolism, and stress responses (Lindemose et al., 2013; Ambawat et al., 2013). Particularly relevant to this study, the MYB-SHAQKYF subfamily members *MYS1* and *MYS2* have been identified as positive regulators of cuticular wax biosynthesis in *Arabidopsis*, functioning through transcriptional suppression of the wax biosynthesis repressor *DEWAX* (Liu et al., 2022). Three 1R-MYB transcription factors (*MYBS1*, *MYBS2*, *MYBS3*) have been characterized in rice, where they mediate sugar and hormone signaling by regulating α-amylase gene expression (Lu et al., 2002). *OsMYBS3* specifically participates in cold stress responses by activating starch hydrolysis to provide carbon sources under low-temperature conditions (Su et al., 2010). A recent study demonstrated that *SiMYBS3* from foxtail millet (*Setaria italica*) enhances drought tolerance when heterologously expressed in *Arabidopsis* (Liu et al., 2023b). However, whether *MYBS3* orthologs function in drought tolerance in monocotyledonous turfgrass species remains unknown.

Building on the above studies, several fundamental questions remain unanswered in Kentucky bluegrass: (i) whether cultivar-level variation in cuticular wax density correlates with drought performance; (ii) whether comparative transcriptomics can identify candidate transcription factors whose expression profiles track both the inter-cultivar wax gradient and stress-induction dynamics; and (iii) whether *PpMYBS3*, a 1R-MYB transcription factor, is sufficient to enhance drought-associated traits through modulation of cuticular wax-related epidermal structures. To address these questions, we conducted integrated phenotypic characterization (including quantitative SEM analysis, physiological measurements of RWC, MDA, antioxidant enzymes, and ROS staining), *de novo* transcriptomic profiling with rigorous assembly quality control (BUSCO evaluation, redundancy collapse, and read-mapping rate assessment), and stable genetic transformation of *PpMYBS3* in Kentucky bluegrass. This research provides new insights into the molecular regulation of drought-associated epidermal and root traits in Kentucky bluegrass and identifies *PpMYBS3* as a promising candidate for genetic improvement of cool-season turfgrass drought resilience.

## 2 Materials and methods

### 2.1 Plant materials and experimental design

Three Kentucky bluegrass varieties, ‘K.B.G’, ‘Jenny’, and ‘Arcadia’, were obtained from Zhengdao Ecological Technology Co., Ltd. (Beijing, China). Plants were cultivated at the Qiqihar University Nursery Greenhouse (Qiqihar, Heilongjiang Province, China) in nutrient-rich soil composed of loam, sand, and vermiculite (3:1:1, v/v/v). Plants were maintained under natural light conditions with regular irrigation every two days.

For drought stress experiments, uniformly grown plants at the three-leaf stage were transplanted into 10 × 10 × 10 cm pots with uniform substrate. After a two-week acclimation period, drought stress was imposed by withholding water for 20 days, followed by a 7-day recovery period with resumed irrigation. Morphological observations were recorded at 0, 20, and 27 days (7 days post-rewatering).

For transgenic plant analysis, wild-type and *PpMYBS3*-overexpressing plants were subjected to simulated drought stress using 1/2 Hoagland nutrient solution supplemented with 10% PEG6000 (polyethylene glycol). Leaf samples were collected at 0, 2, and 16 h post-treatment, immediately frozen in liquid nitrogen, and stored at −80 °C for subsequent analyses.

### 2.2 Morphological and chlorophyll measurements

Plant height was measured using a ruler, and leaf width and thickness were determined using digital vernier calipers. Chlorophyll extraction was performed following the protocol of Wang et al. (2023). Briefly, 0.2 g of fresh leaf tissue was immersed in 20 mL of 95% ethanol and incubated at room temperature in darkness for 48 h until complete decolorization. Absorbance was measured at 645 nm and 663 nm using a spectrophotometer. Total chlorophyll content was calculated as: Chl=0.04×(20.21×D645+8.02×D663). All measurements were performed with three biological replicates and three technical replicates.

### 2.3 Paraffin sectioning and anatomical analysis

Fresh leaves were cut into 2 cm segments and fixed in FAA solution (formalin:acetic acid:70% ethanol, 5:5:90, v/v/v) for 48 h. Samples were dehydrated through an ethanol gradient (70%, 80%, 85%, 90%, 95%, and 100%), cleared in xylene, and embedded in paraffin at 70 °C. Sections (8 μm) were prepared using a rotary microtome, stained with safranin-fast green, and mounted with Canada balsam. Images were captured using a light microscope (Olympus BX51, Japan).

### 2.4 Scanning electron microscopy

SEM observation was performed following the methodology of Jiang et al. (2024). Fresh leaf segments (2 mm³) were washed three times with 0.1 M phosphate buffer and fixed overnight in 2.5% glutaraldehyde at 4 °C, followed by post-fixation in 1% osmium tetroxide for 1–2 h. Samples were dehydrated through an ethanol series (30%, 50%, 70%, 80%, 90%, 95%, 100%), critical-point dried, and sputter-coated with gold using an ion sputtering device (IXRF 550i). Images were captured using a scanning electron microscope (HITACHI SU8010, Japan).

### 2.5 RNA extraction and transcriptome sequencing

Total RNA was extracted from leaves using QIAzol Lysis Reagent (Qiagen, Germany). RNA quality was assessed using an Agilent 5300 Bioanalyzer, and quantity was determined using a NanoDrop ND-2000 spectrophotometer. RNA-seq libraries were constructed using the Illumina Stranded mRNA Prep Ligation kit with 1 μg total RNA input. Paired-end sequencing (PE150) was performed on the NovaSeq X Plus platform at Shanghai Majorbio Bio-pharm Biotechnology Co., Ltd. (Shanghai, China).

### 2.6 *De Novo* transcriptome assembly and annotation

Raw reads were trimmed and quality-controlled using fastp v0.23.2 with default parameters (Chen et al., 2018). To maximize transcript discovery across all three cultivars, clean reads from all nine libraries were pooled and assembled jointly using Trinity v2.14.0 (Grabherr et al., 2011) with default parameters (k-mer size = 25, minimum contig length = 200 bp). This pooled assembly approach is recommended for multi-genotype comparisons in species lacking a reference genome (Haas et al., 2013), as it captures both shared and cultivar-specific transcripts in a single reference.

To mitigate chimeric assembly artifacts arising from homeologous sequences and inter-cultivar polymorphisms, we applied the following quality-control measures. First, redundant transcripts were collapsed using CD-HIT-EST v4.8.1 (Fu et al., 2012) at a 95% sequence identity threshold; the longest representative sequence per cluster was retained as the “unigene,” reducing the total from 356822 transcripts to 228310 unigenes (mean length 740 bp; N50–1073 bp; **Supplementary Table 3**). The 95% identity threshold was selected to collapse allelic variants and very recent duplicates while preserving homeologs that have diverged beyond this threshold—a standard practice for polyploid *de novo* assemblies (Krasileva et al., 2013). Second, assembly structural integrity was evaluated using TransRate v1.0.3 (Smith-Unna et al., 2016), which assesses per-contig evidence of chimeric structure based on read-pair mapping metrics. The analysis classified 78.3% of contigs as structurally well-supported (“good” contigs), with a TransRate assembly score of 0.21, comparable to published *de novo* assemblies of polyploid grasses (**Supplementary Table 3**). Third, assembly completeness was evaluated using BUSCO v5.4.7 (Simao et al., 2015) against the Poales_odb10 lineage dataset; the assembly captured 76.8% complete BUSCOs (74.6% single-copy, 2.2% duplicated) (**Supplementary Table 3**). This completeness level is consistent with published *de novo* transcriptome assemblies of polyploid grasses sampled from single tissues: Bushman et al. (2021) reported 67.4% in *P. pratensis*; Byrne et al. (2015) reported ~71% in tetraploid *Lolium perenne*; and Huang et al. (2020) reported 73.2% in tetraploid *Dactylis glomerata*. The moderate BUSCO completeness reflects the inherent limitation that not all conserved orthologs are expressed in leaf tissue under a single developmental condition, rather than fundamental assembly deficiency. The average read-mapping rate back to the assembly was 82.7 ± 2.3% across nine libraries (**Supplementary Table 2**).

Unigenes were annotated against NCBI non-redundant protein (NR), COG, KEGG, GO, Pfam, and Swiss-Prot databases using DIAMOND BLASTX with an E-value threshold< 1.0 × 10^-5^. GO annotations were obtained using BLAST2GO (Conesa et al., 2005).

The high unigene count (228310) is attributable to three factors: the high ploidy of Kentucky bluegrass (2n = 4x–8x ≈ 28–56) (Bushman et al., 2013), which generates homeologous transcripts diverging beyond the 95% CD-HIT-EST threshold; the pooling of three genetically distinct cultivars, which introduces inter-cultivar allelic diversity; and the inherent tendency of *de novo* assemblers to fragment lowly expressed transcripts. Comparable or higher counts have been reported in polyploid Poaceae: ~145000 unigenes from a single cultivar of *P. pratensis* (Bushman et al., 2021); ~200000 transcripts in tetraploid *Lolium perenne* (Byrne et al., 2015); and >300000 transcripts in hexaploid *Triticum aestivum* (Krasileva et al., 2013; Clavijo et al., 2017). Our three-cultivar pooled count of 228310 is proportionally consistent with these benchmarks. We note that only annotated unigenes (80366 with GO annotation; 47645 with KEGG annotation) were used for functional enrichment analyses, and the candidate gene *PpMYBS3* was independently validated by qRT-PCR and transgenic overexpression, ensuring that biological conclusions are not contingent on assembly completeness alone.

### 2.7 Differential expression analysis

Gene expression was quantified at the gene level using RSEM v1.3.3 (Li and Dewey, 2011) operating in Trinity mode (--trinity-mode), which utilizes the isoform-to-gene mapping file (Trinity.fasta.gene_trans_map) automatically generated by Trinity. In this framework, each Trinity “component” (identified by the shared TRINITY_DNxxxxxgx prefix) represents a putative gene locus grouping all isoforms assembled from the same de Bruijn graph component. RSEM employs an expectation-maximization algorithm to allocate multi-mapping reads among isoforms and then aggregates isoform-level estimates to produce gene-level counts and TPM values (Haas et al., 2013). This approach is the standard quantification workflow for *de novo* Trinity assemblies and avoids inflation of feature counts that would result from treating each isoform as an independent gene.

Gene-level count matrices were extracted using Trinity’s abundance_estimates_to_matrix.pl utility. Differential expression analysis was conducted using DESeq2 v1.36.0 (Love et al., 2014) on gene-level counts. Genes with |log_2_FC| ≥ 1 and FDR< 0.05 were defined as differentially expressed genes (DEGs). GO and KEGG enrichment analyses were performed with Bonferroni-corrected *P*-value< 0.05.

### 2.8 Subcellular localization and genetic transformation

The full-length *PpMYBS3* coding sequence was cloned into the pCAMBIA1300-eGFP vector at KpnI and BamHI restriction sites. The construct was transformed into *Agrobacterium tumefaciens* GV3101 and infiltrated into *Nicotiana tabacum* leaves for transient expression. Subcellular localization was observed using a laser scanning confocal microscope 48 h post-infiltration.

For stable transformation, the optimized protocol of Sun et al. (2022) was employed using ‘Jenny’ as the transformation recipient. A total of six independent T0 lines were regenerated, of which three were confirmed as positive transformants by genomic PCR and qRT-PCR. Two independent lines (OE-3 and OE-5) showing the highest overexpression levels were selected for all subsequent phenotypic analyses. All quantitative data represent the mean of both independent lines, each with three biological replicates (n = 3 per line, total n = 6 per genotype). This design ensures that observed phenotypes are attributable to *PpMYBS3* overexpression rather than positional effects of transgene insertion. The primer pairs used for positive-line screening and expression quantification are listed in **Supplementary Table 6**.

### 2.9 Root architecture analysis

Root system architecture of 9-week-old wild-type and transgenic plants was analyzed using a ScanMaker i800 Plus root scanner. Total root number, root length, surface area, and volume were quantified using WinRHIZO software (Regent Instruments, Canada). Three biological replicates per genotype were analyzed.

### 2.10 DAB and NBT staining

Reactive oxygen species (ROS) accumulation was visualized using 3,3’-diaminobenzidine (DAB) staining for H_2_O_2_ and nitroblue tetrazolium (NBT) staining for superoxide (O_2_⁻) according to manufacturer’s instructions (Coolaber, China). Leaf segments (3 cm) were vacuum-infiltrated with staining solution, incubated overnight at room temperature, and decolorized in 95% ethanol at 80 °C.

### 2.11 Quantitative real-time PCR

Total RNA was extracted using the RNAprep Pure Plant Kit (TIANGEN, China) and reverse-transcribed using the HiFiScript cDNA Synthesis Kit (CWBIO, China). qRT-PCR was performed using TB Green Premix Ex Taq II (Takara, Japan) on a CFX96 Real-Time PCR Detection System (Bio-Rad, USA). Thermal cycling conditions were: 95°C for 30 s, followed by 40 cycles of 95°C for 5 s and 60°C for 30 s. The *Poa pratensis UBQ* gene was used as the internal reference gene for normalization. Relative expression levels were calculated using the 2^-ΔΔCt^ method (Livak and Schmittgen, 2001). Each experiment included three biological replicates with three technical replicates per sample. Primer sequences are listed in **Supplementary Table 6**.

### 2.12 Statistical analysis

Statistical analyses were performed using SPSS v10.0 (SPSS Inc., Chicago, IL, USA). Data from multiple groups were analyzed by one-way analysis of variance (ANOVA) followed by Tukey’s honestly significant difference (HSD) test for multiple comparisons. For pairwise comparisons, Student’s *t*-test was applied. Statistical significance was set at *P* < 0.05. Data are presented as mean ± standard deviation (SD) from at least three biological replicates (n ≥ 3). ImageJ was used for quantification of papillae volume and stomatal area. Graphs were generated using GraphPad Prism v9.00 and TBtools.

## 3 Results

### 3.1 Phenotypic and anatomical characterization reveals morphological variation among Kentucky bluegrass cultivars

**Figure 1:**
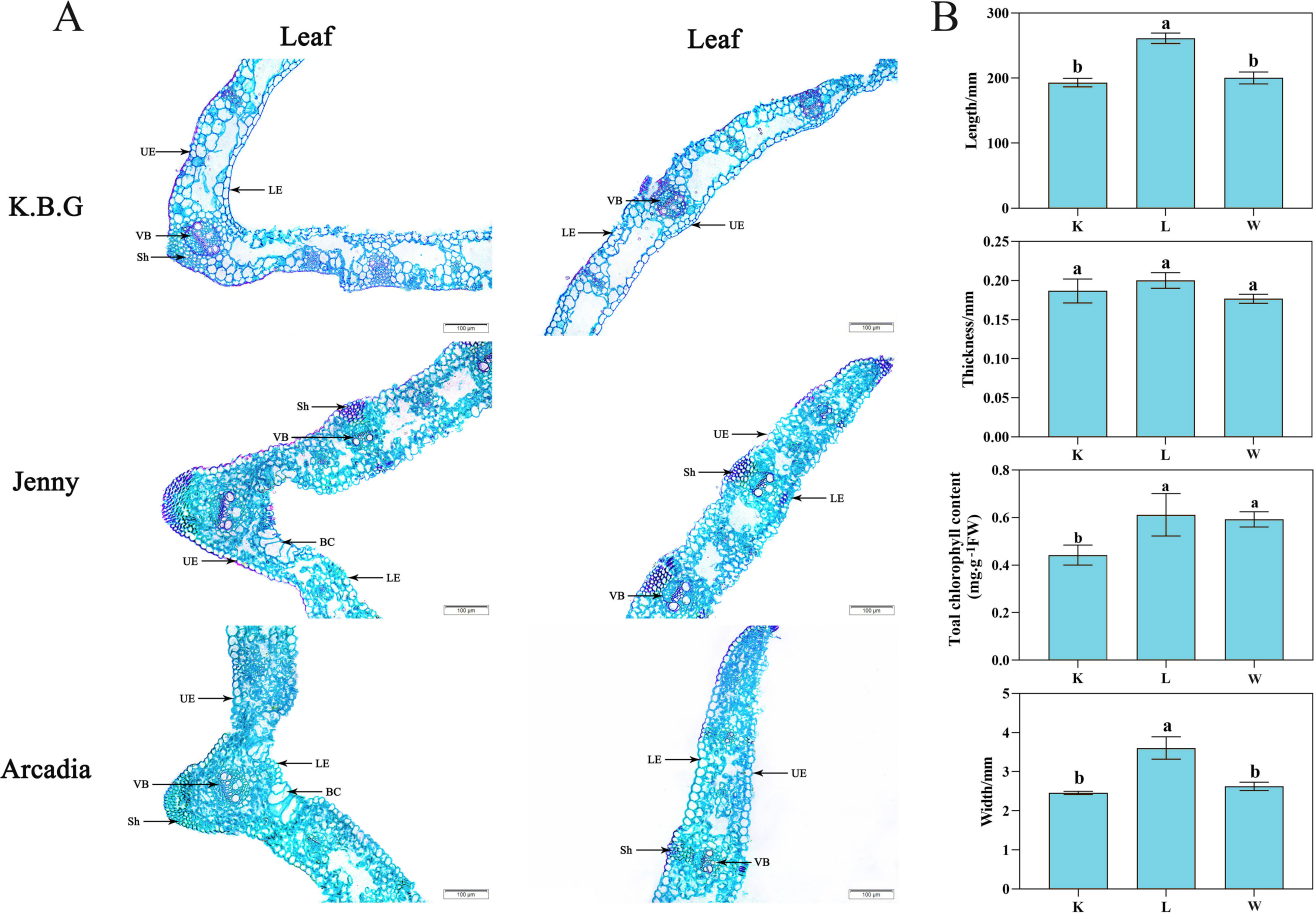
Anatomical structure and phenotypic analysis of three Kentucky bluegrass cultivars. **(A)** Light microscopy observation of leaf cross-section anatomy for K.B.G, Jenny, and Arcadia (UE, upper epidermis; LE, lower epidermis; VB, vascular bundle; SH, sclerenchyma; BC, bulliform cells). Scale bar = 100 μm. **(B)** Phenotypic measurements of the three cultivars: length (mm), leaf width (mm), leaf thickness (mm), and total chlorophyll content (mg·g⁻¹ FW). K, L and W respectively represent K.B.G, Arcadia and Jenny. Values represent mean ± SD (n = 3). Different letters indicate significant differences at *P* < 0.05 (Tukey’s HSD).

To establish the phenotypic foundation for subsequent functional analyses, we first characterized the morphological and anatomical features of three Kentucky bluegrass cultivars: K.B.G, Jenny, and Arcadia (**Figure 1**). Significant inter-cultivar differences were observed in leaf dimensions. Arcadia exhibited significantly longer (*P* < 0.05) and wider leaves compared to K.B.G and Jenny, whereas leaf thickness showed no significant variation among cultivars (**Figure 1B**). Total chlorophyll content (mg·g^-1^ FW) in K.B.G was significantly lower than in Arcadia and Jenny (*P* < 0.05; **Figure 1B**).

Anatomical examination of leaf cross-sections revealed that all three cultivars possessed the typical monocot leaf structure comprising upper and lower epidermis, vascular bundles, and sclerenchyma (**Figure 1A**). To quantify the observed differences in mesophyll organization, mesophyll cell area was measured from paraffin cross-sections using ImageJ (n = 30 cells per cultivar, three biological replicates). K.B.G exhibited significantly larger mesophyll cells (1847 ± 213 μm^2^) compared to Jenny (1124 ± 187 μm^2^) and Arcadia (1089 ± 162 μm^2^; *P* < 0.05, Tukey’s HSD; **Supplementary Figure 1A**), indicating that the denser mesophyll packing in Jenny and Arcadia reflects smaller cell size rather than merely altered spacing.

### 3.2 Cuticular wax-associated epidermal structures vary significantly among cultivars

**Figure 2:**
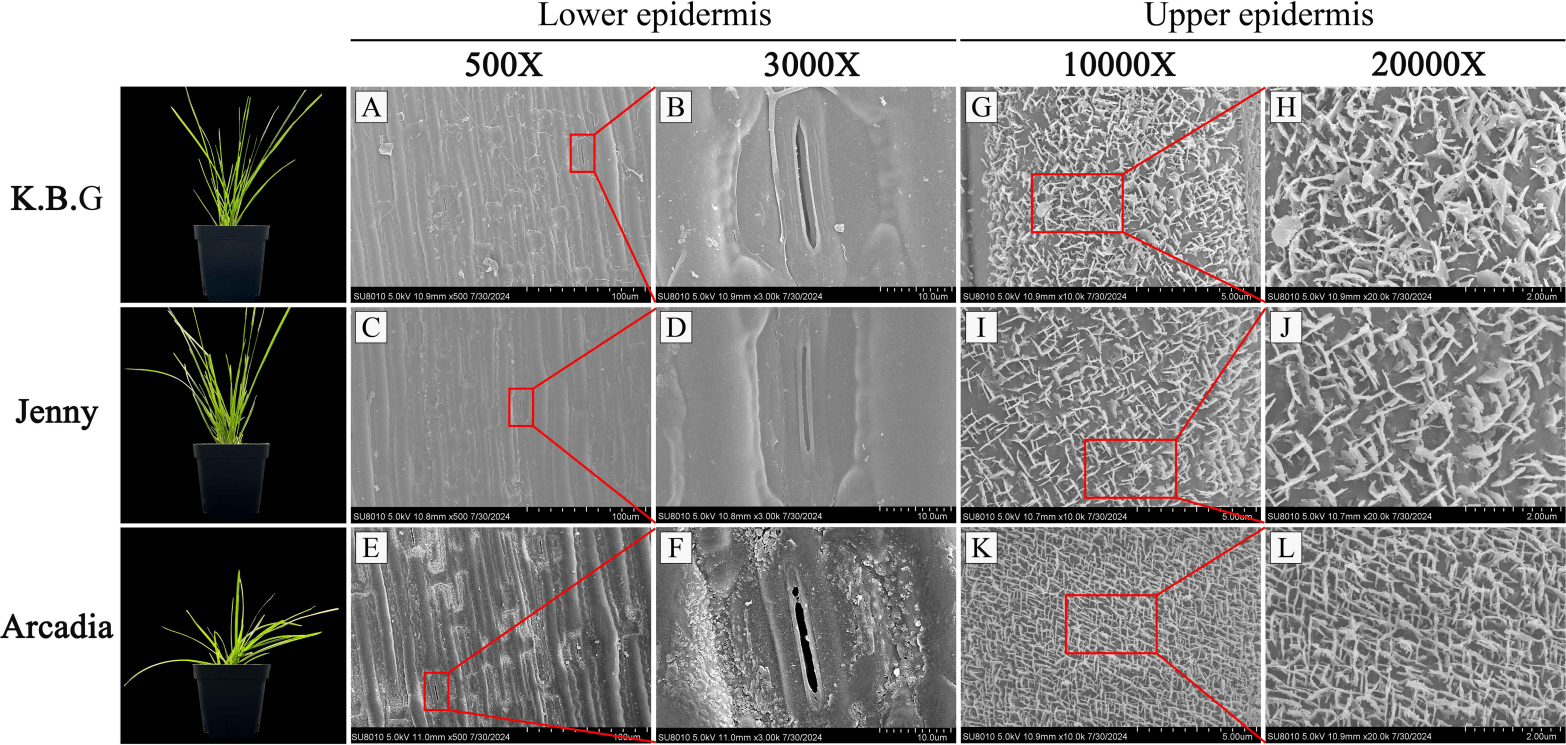
SEM images of upper and lower epidermis of leaves of K.B.G, Arcadia and Jenny. **(A–F)** Ultrastructure of lower epidermis of leaves of three Kentucky bluegrass varieties. **(G–L)** Ultrastructure of upper epidermis of leaves of three Kentucky bluegrass varieties.

Given the established role of cuticular wax in drought protection, we employed scanning electron microscopy (SEM) to characterize the ultrastructure of leaf epidermal surfaces across the three varieties (**Figure 2**). Substantial inter-varietal differences in wax crystal density and distribution were observed on both adaxial and abaxial leaf surfaces.

On the abaxial (lower) epidermis, K.B.G and Jenny displayed minimal wax crystal deposition (**Figures 2A–D**), whereas Arcadia exhibited detectable wax crystals surrounding stomata (**Figures 2E, F**). On the adaxial (upper) epidermis, the differences were more pronounced. To provide quantitative support for these observations, wax crystal density was determined from SEM images at 10000× magnification using ImageJ particle counting (n = 5 fields per cultivar per leaf surface). On the adaxial surface, Arcadia displayed the highest wax crystal density (38.2 ± 5.1 crystals per 100 μm^2^), followed by K.B.G (21.7 ± 4.3 crystals per 100 μm^2^), and Jenny exhibited the lowest density (8.4 ± 2.6 crystals per 100 μm^2^; all pairwise comparisons *P* < 0.05, Tukey’s HSD; **Supplementary Figure 1B**). These quantitative data establish a clear gradient of wax-associated epidermal structures among cultivars (Arcadia > K.B.G > Jenny).

We note that SEM-based crystal density provides morphological evidence of wax-associated structures but does not directly quantify total wax load or wax composition. Future studies employing GC-MS-based wax profiling and cuticular transpiration assays will be necessary to establish the biochemical basis and functional significance of these structural differences.

### 3.3 Cultivar variation in wax-associated epidermal structures co-varies with stress-related phenotypes

**Figure 3:**
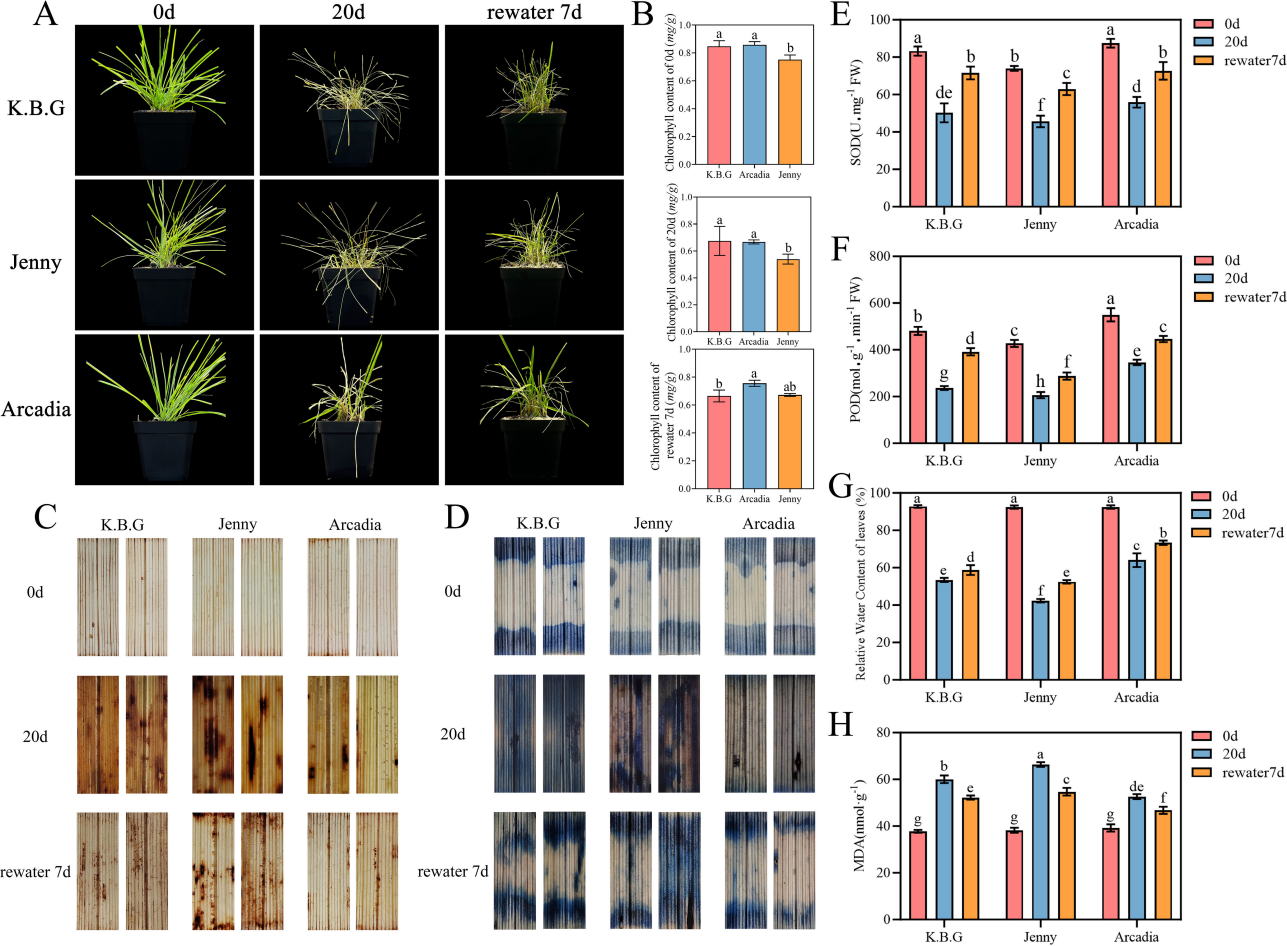
Drought tolerance assessment of three Kentucky bluegrass cultivars. **(A)** Morphological appearance of K.B.G, Arcadia, and Jenny at 0 d, 20 d (drought), and 27 d (7 d post-rewatering). **(B)** Chlorophyll content (mg/g). **(C)** DAB staining (H_2_O_2_) at 0 d, 20 d, and 7 d post-rewatering. **(D)** NBT staining (O_2_⁻) at the same time points. **(E)** SOD activity (U·g⁻¹ FW). **(F)** POD activity (U·g⁻¹ FW). **(G)** Relative water content (%). **(H)** MDA content (nmol·g⁻¹ FW). Different letters indicate significant differences (*P<* 0.05, Tukey’s HSD). Values represent mean ± SD (n = 3).

To examine whether cultivar differences in wax-associated epidermal structures relate to drought-related performance, plants were subjected to a pot withholding assay (20 d without irrigation) followed by rewatering. Cultivars displayed distinct injury phenotypes during stress, To quantify these visual differences, a semi-quantitative leaf wilting score was applied (0 = fully turgid, 5 = completely desiccated) modified from Bushman et al. (2021) and assessed independently by three evaluators. At day 20, Jenny received the highest injury score (4.3 ± 0.5), followed by K.B.G (3.0 ± 0.6) and Arcadia (1.8 ± 0.4; *P* < 0.05, ANOVA; **Supplementary Table 1**). After rewatering, recovery scores also differed significantly, with Arcadia showing the most rapid regrowth (**Figure 3B**; **Supplementary Table 1**).

Because drought stress is frequently accompanied by oxidative bursts, we assessed ROS accumulation using DAB (H_2_O_2_) and NBT (O_2_^-^) staining. At the end of drought treatment, staining intensity increased in all cultivars relative to the well-watered control, while Arcadia showed consistently weaker staining than Jenny (**Figures 3C, D**). In parallel, SOD and POD activities were measured at days 0 and 20 (**Figures 3E, F**). Notably, SOD and POD activities declined in all cultivars at day 20 relative to day 0, which contrasts with the transient enzymatic increases often reported in short-term drought studies. The chlorophyll content and the rate of chlorophyll decline exhibited a similar trend (**Figure 3B**, **Supplementary Table S1**). This decline is consistent with the well-documented biphasic antioxidant response model (Xu et al., 2011), in which enzyme activities increase during early/moderate stress but decline under prolonged severe stress due to oxidative inactivation of enzyme proteins and substrate depletion. Xu et al. (2011) specifically reported this pattern in Kentucky bluegrass, where SOD activity peaked at approximately 8–12 d of water withholding and subsequently declined. To account for baseline differences among cultivars, we calculated the percentage reduction (Δ%) from day 0 to day 20 for each parameter. The relative decline in chlorophyll content was greatest in Jenny (Δ% = −38.7 ± 4.2%), intermediate in K.B.G (Δ% = −29.1 ± 3.5%), and smallest in Arcadia (Δ% = −18.4 ± 2.8%; all pairwise *P* < 0.05; **Supplementary Table 1**). Similarly, percentage declines in SOD and POD were greatest in Jenny and smallest in Arcadia, consistent with the drought tolerance ranking.

Additionally, we measured relative water content (RWC) and malondialdehyde (MDA) content to provide complementary physiological evidence. At day 20 of drought, RWC was highest in Arcadia (65.3 ± 4.1%), intermediate in K.B.G (51.7 ± 3.2%), and lowest in Jenny (41.4 ± 2.8%; *P* < 0.05; **Figure 3G**). MDA content showed the inverse pattern: Jenny (68.2 ± 4.1 nmol·g>⁻¹ FW) > K.B.G (60.8 ± 3.5) > Arcadia (53.6 ± 2.9; *P* < 0.05; **Figure 3H**), indicative of greater lipid peroxidation in the drought-sensitive cultivar. Together, these observations indicate that cultivar-level variation in wax-associated epidermal structures co-occurs with differences in drought-associated injury, water retention capacity, and oxidative stress responses. Notably, because cultivars also differ in plant size and leaf morphology (**Figure 1**), and because soil water status was not quantified, these results support an association rather than a direct causal inference.

### 3.4 *De novo* transcriptome assembly and differential expression among cultivars

We performed *de novo* RNA-seq of leaf tissues from the three cultivars (three biological replicates per cultivar). After quality filtering using fastp (Chen et al., 2018), 459 million clean reads were retained (**Supplementary Table 2**). Clean reads from all nine libraries were pooled and assembled jointly using Trinity v2.14.0 (Grabherr et al., 2011) with default parameters (k-mer = 25, minimum contig length = 200 bp). A joint assembly strategy was chosen to maximize transcript recovery across all three cultivars, as recommended for multi-genotype comparative studies lacking a reference genome (Real et al., 2020). Redundant transcripts were collapsed using CD-HIT-EST v4.8.1 (Fu et al., 2012) at a 95% sequence identity threshold, reducing the total from 356822 transcripts to 228310 unigenes (mean length 740 bp; N50–1073 bp; **Supplementary Table 3**).

To assess potential chimeric artifacts-a known concern when pooling reads from multiple genotypes of a polyploid species-we evaluated assembly structural quality using TransRate v1.0.3 (Smith-Unna et al., 2016). The analysis classified 78.3% of assembled contigs as structurally supported based on read-pair mapping consistency (TransRate assembly score = 0.21; **Supplementary Table 3**), indicating that the majority of contigs do not exhibit signatures of chimeric mis-assembly. Assembly completeness was assessed by BUSCO v5.4.7 against the Poales_odb10 lineage dataset, yielding 76.8% complete BUSCOs (74.6% single-copy, 2.2% duplicated) (**Supplementary Table 3**). The low duplicated BUSCO fraction (2.2%) indicates that homeologous copies of conserved genes were largely resolved into separate unigenes rather than collapsed, consistent with the expected behavior for a high-ploidy species. The average read-mapping rate was 82.7 ± 2.3% across all nine libraries (**Supplementary Table 2**). A total of 80366 unigenes were annotated in GO and 47645 in KEGG (**Supplementary Table 4**). Most annotated sequences matched grass species, consistent with the phylogenetic background of *Poa*.

The high unigene count is consistent with the known polyploid nature of Kentucky bluegrass (2n = 4x–8x ≈ 28–56) (Bushman et al., 2013), the pooling of three genetically distinct cultivars, and the inherent transcript fragmentation of short-read *de novo* assembly. Comparable *de novo* assemblies in other polyploid Poaceae have reported similar magnitudes: ~145000 unigenes in *P. pratensis* from a single cultivar (Bushman et al., 2021); ~200000 in tetraploid *Lolium perenne* (Byrne et al., 2015); and >300000 in *hexaploid Triticum aestivum* (Krasileva et al., 2013; Clavijo et al., 2017).

Using |log_2_FC| ≥ 1 and FDR< 0.05, differential expression analysis was performed at the gene level (Trinity component level) using RSEM for quantification (with the --trinity-mode flag to leverage the inherent isoform-to-gene mapping; see Section 2.7) and DESeq2 for statistical testing, thereby grouping isoforms from the same gene locus and avoiding inflation of DEG counts. Pairwise comparisons identified 29713 DEGs (K.B.G vs. Arcadia), 7257 DEGs (K.B.G vs. Jenny), and 21149 DEGs (Arcadia vs. Jenny) (**Supplementary Table 5**). These results indicate substantial transcriptomic divergence among cultivars under the sampled conditions.

### 3.5 Pathway enrichment suggests hormone signaling differences among cultivars

**Figure 4:**
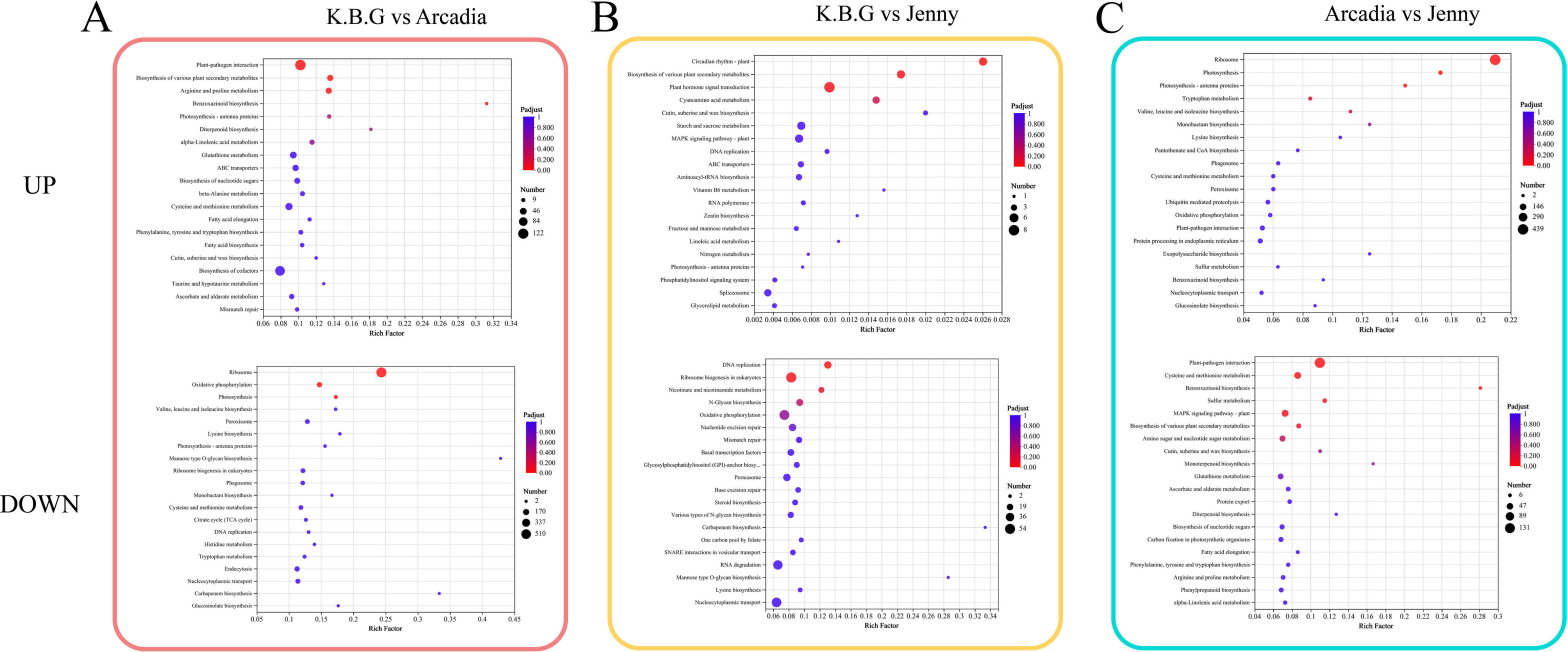
KEGG pathway enrichment analysis of differentially expressed genes (DEGs) among three Kentucky bluegrass cultivars. **(A)** K.B.G vs. Arcadia; **(B)** K.B.G vs. Jenny; **(C)** Arcadia vs. Jenny. Bubble size represents the number of DEGs enriched in each pathway; the color gradient indicates -log_10_ (adjusted *P*-value); the x-axis represents the Rich Factor (number of DEGs in a pathway/total number of annotated genes in that pathway). “Plant hormone signal transduction” is highlighted as the recurrently enriched pathway across comparisons.

KEGG enrichment of DEGs highlighted “plant hormone signal transduction” across multiple comparisons (**Figure 4**), suggesting that cultivar divergence may involve differences in stress-related signaling modules. Given that hormone pathways can influence stomatal behavior, root development, and epidermal traits, we next examined the expression patterns of representative components within auxin, ABA, and SA signaling pathways.

### 3.6 Expression differences in auxin/ABA/SA signaling components

**Figure 5:**
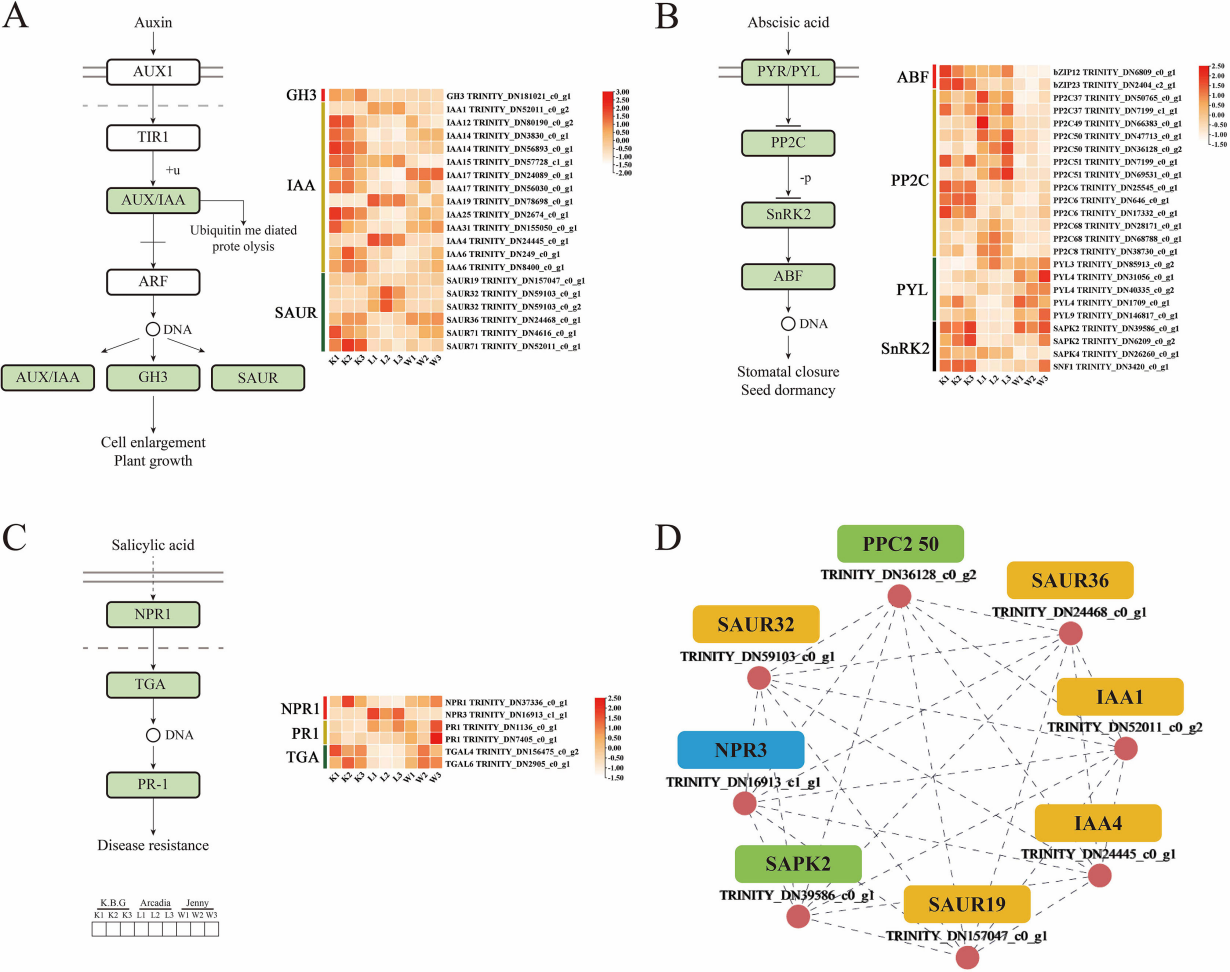
Analysis of plant hormone signal transduction pathways in Kentucky bluegrass. **(A)** Analysis of auxin signaling pathway and its differential gene expression. **(B)** Analysis of abscisic acid signaling pathway and its differential gene expression. **(C)** Analysis of salicylic acid signaling pathway and its differential gene expression. **(D)** Correlation analysis of key differential genes in auxin, abscisic acid and salicylic acid signaling pathways.

Within the auxin signaling module, several Aux/IAA, GH3, and SAUR family members displayed cultivar-dependent expression patterns (**Figure 5A**). In the ABA signaling module, transcripts annotated as PYR/PYL receptors, PP2Cs, SnRK2s, and ABFs also varied across cultivars (**Figure 5B**). SA signaling components (e.g., NPR1/NPR3 and TGA factors) showed differential patterns as well (**Figure 5C**). While these expression profiles are consistent with cultivar-dependent signaling states, transcript abundance alone does not establish pathway activity; quantification of hormone levels and/or downstream physiological readouts (e.g., stomatal conductance, ABA sensitivity assays) will be required to determine functional consequences. Correlation analysis among selected genes (**Figure 5D**) is presented as a co-expression summary and should be interpreted as hypothesis-generating rather than evidence of regulatory relationships.

### 3.7 Identification and expression analysis of MYB transcription factor family genes

**Figure 6:**
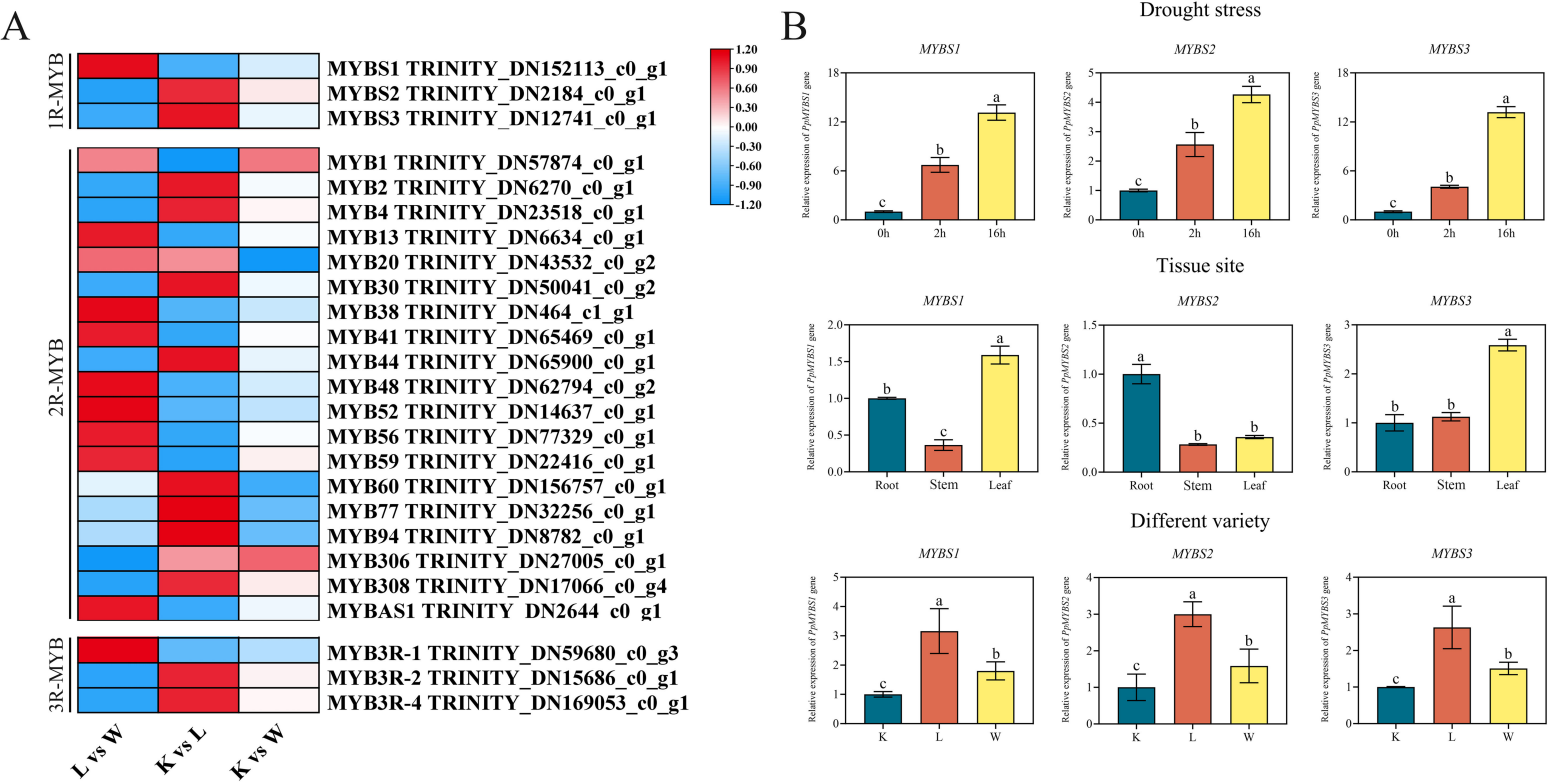
Expression analysis of MYB transcription factor family genes. **(A)** Heatmap of RNA-seq expression levels (TPM) for 1R-MYB, 2R-MYB, and 3R-MYB subfamily members across K.B.G, Arcadia, and Jenny (diverging blue – white – red scheme; blue = low, white = median, red = high). **(B)** qRT-PCR validation of 1R-MYB genes (*MYBS1*, *MYBS2*, *MYBS3*). Upper panel: osmotic-stress time course (0, 2, 16 h). Middle panel: tissue-specific expression in Arcadia (root, stem, leaf). Lower panel: cross-cultivar comparison in leaf tissue (K.B.G, Arcadia, Jenny), where K.B.G was used as the calibrator (set to 1.0). K, L and W represent K.B.G, Arcadia and Jenny, respectively. *PpUBQ* was used as the internal reference. Values represent mean ± SD (n = 3). Different letters indicate significant differences (*P* < 0.05).

To identify transcriptional regulators potentially involved in drought tolerance and wax-related epidermal traits, we examined the MYB transcription factor family in the *de novo* transcriptome and compared their expression across cultivars. RNA-seq expression was quantified at the gene level (Trinity component/cluster) as TPM values. For heatmap visualization (**Figure 6A**), TPM values were log-transformed and displayed using a diverging blue–white–red scale to distinguish lower and higher expression. We note that heatmap visualization emphasizes relative differences among cultivars for each gene and, depending on scaling, is not intended to be interpreted as absolute transcript abundance.

To prioritize candidates for functional testing, we applied a multi-criteria filtering strategy. First, MYB-family DEGs were defined by Pfam annotation of the MYB DNA-binding domain (PF00249), yielding 87 MYB-family DEGs. Second, based on functional precedent for 1R-MYB/SHAQKYF members in epidermal wax regulation and abiotic stress responses (Liu et al., 2022, Liu et al., 2023b), we focused on the 1R-MYB subgroup, which contained three members (*PpMYBS1*, *PpMYBS2*, and *PpMYBS3*). Third, among these, *PpMYBS3* showed the strongest concordance with the cultivar wax-crystal density gradient (Arcadia > K.B.G > Jenny) and exhibited the largest differential expression in the Arcadia vs. Jenny RNA-seq comparison (|log2FC| = 3.42, FDR = 2.1 × 10^⁻8^). Fourth, qRT-PCR confirmed that *PpMYBS3* was stress inducible and showed robust induction dynamics relative to the other two 1R-MYBs. Fifth, phylogenetic analysis placed *PpMYBS3* closest to *OsMYBS3*/*SiMYBS3*, which have documented stress-protective functions (Su et al., 2010; Liu et al., 2023b).

For qRT-PCR, relative expression was calculated using the 2^⁻ΔΔCt^ method with *PpUBQ* as the internal reference. Importantly, for the cross-cultivar comparison (lower panel of **Figure 6B**), K.B.G was used as the calibrator and set to 1.0; therefore, values for Arcadia and Jenny represent fold changes relative to K.B.G rather than absolute abundance. In addition, the RNA-seq heatmap (**Figure 6A**) and qRT-PCR plots (**Figure 6B**) differ in normalization (TPM vs. 2^⁻ΔΔCt^) and in the exact experimental context (single-condition transcriptome sampling vs. targeted validation across treatments/tissues). Thus, complete visual identity of expression patterns between panels is not expected; instead, we interpret consistency in directionality (e.g., stress inducibility) and cultivar ranking as the primary validation criterion. Notably, Arcadia showed higher relative expression of *PpMYBS2* and *PpMYBS3* than K.B.G under the tested condition (K.B.G = 1.0), consistent with Arcadia exhibiting the highest wax-crystal density. Based on the convergence of these criteria, *PpMYBS3* was selected for downstream functional characterization.

### 3.8 Subcellular localization and transgenic validation of *PpMYBS3*

**Figure 7:**
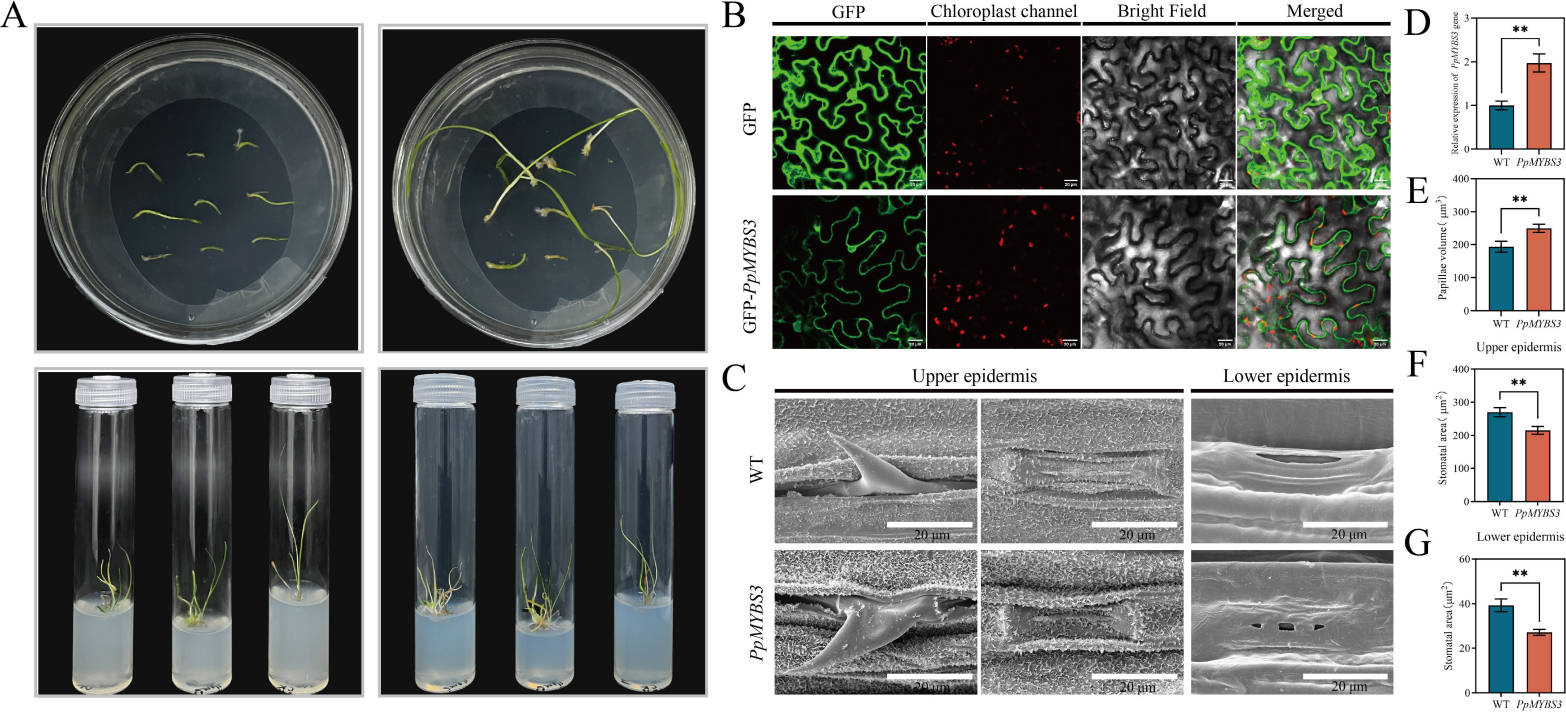
Acquisition of transgenic plants, SEM and subcellular localization analysis. **(A)** Acquisition of positive plants. **(B)** Subcellular localization analysis of PpMYBS3 gene. **(C)** SEM observation. **(D)** Overexpression Analysis of PpMYBS3. **(E)** Papillae volume. **(F)** Stomatal area of upper epidermis. **(G)** Stomatal area of lower epidermis. Values represent mean±SD (n=3). ** indicate significant differences (*P* < 0.05).

Based on its expression pattern correlating with drought tolerance and the multi-criteria prioritization described above, *PpMYBS3* was selected for functional characterization. Subcellular localization analysis using *PpMYBS3*-GFP fusion protein in tobacco epidermal cells demonstrated that *PpMYBS3* localizes to both the nucleus and cytoplasm (**Figure 7B**), consistent with its putative function as a transcription factor.

To functionally validate *PpMYBS3*, we generated stable transgenic Kentucky bluegrass lines overexpressing *PpMYBS3* (**Figure 7A**). A total of six independent T0 transgenic lines were regenerated, of which three were confirmed as positive transformants by genomic PCR and qRT-PCR. Two independent lines (OE-3 and OE-5) showing the highest overexpression levels were selected for phenotypic characterization (**Supplementary Figure 2**). qRT-PCR confirmed that *PpMYBS3* expression in transgenic lines was higher than in wild-type plants (*P* < 0.05; **Figure 7D**).

SEM analysis of transgenic plants revealed substantial morphological differences in leaf epidermal ultrastructure compared to wild-type plants (**Figure 7C**). Transgenic lines exhibited increased wax-associated epidermal structures and enlarged papillae on the leaf surface. Quantitative analysis demonstrated that papillae volume in transgenic plants was 1.2-fold that of wild-type (*P* < 0.05; **Figure 7E**). Notably, stomatal aperture area was significantly reduced in transgenic plants: upper epidermis stomatal area was 0.79-fold (20% reduction) and lower epidermis stomatal area was 0.87-fold (13% reduction) compared to wild-type plants (*P* < 0.05; **Figures 7F, G**). These ultrastructural modifications indicate that *PpMYBS3* overexpression promotes wax-associated epidermal structures and may contribute to reduced water loss through decreased stomatal aperture.

To provide complementary molecular evidence supporting a role for *PpMYBS3* in the wax biosynthetic pathway, we performed qRT-PCR analysis of canonical wax biosynthesis and transport genes in wild-type versus *PpMYBS3*-overexpressing lines (**Supplementary Figure 3**; **Supplementary Table 6**). Transcripts of *CER1* (alkane-forming pathway), *KCS6* (very-long-chain fatty acid elongation), and *ABCG11* (wax transporter) were significantly upregulated in overexpression lines relative to wild-type (*P* < 0.05), while *LACS2* (long-chain acyl-CoA synthetase) showed non-significant trends toward regulation. These results are consistent with *PpMYBS3* acting as a positive transcriptional regulator of at least a subset of wax biosynthesis pathway genes, although direct binding evidence (e.g., ChIP-qPCR or EMSA) will be required to confirm direct target relationships.

### 3.9 *PpMYBS3* overexpression enhances root system development

**Figure 8:**
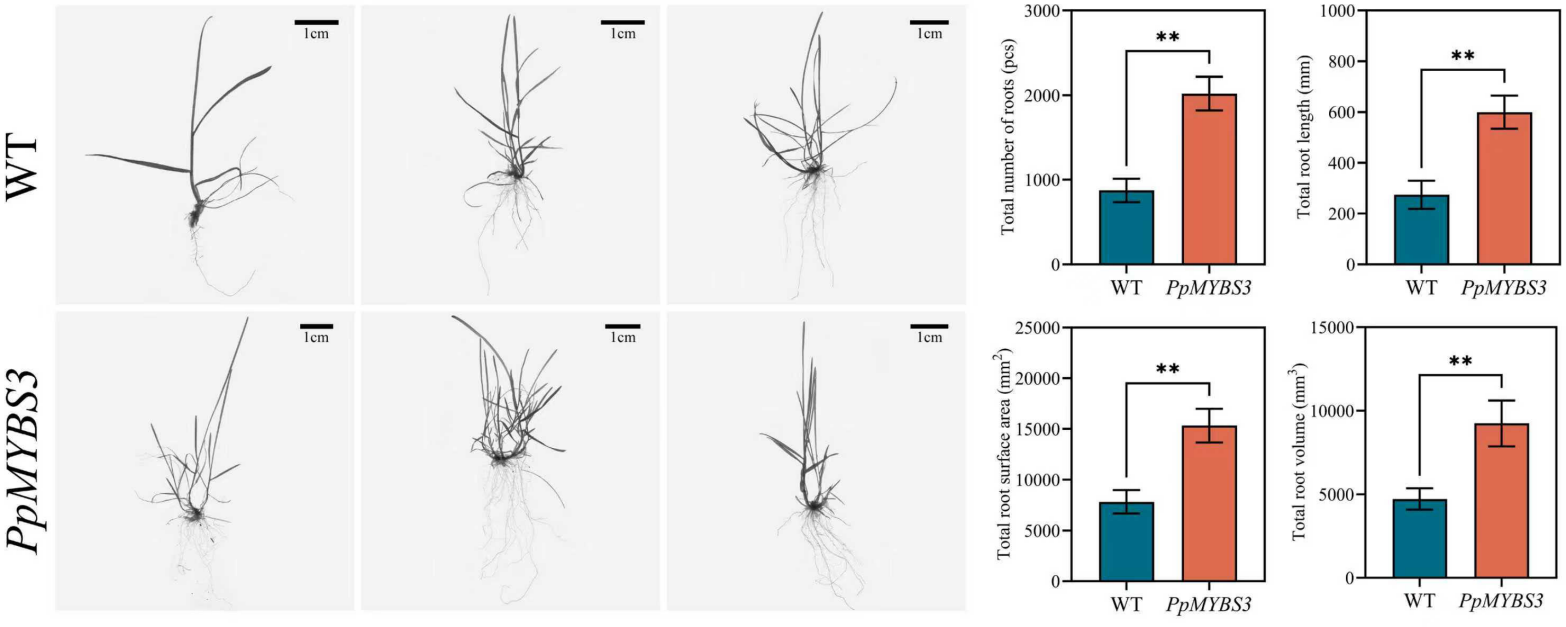
Impact of overexpressed PpMYBS3 on root development in Kentucky Bluegrass. Values represent mean±SD (n=3). ** indicate significant differences (P < 0.05).

Root system architecture was analyzed in wild-type and transgenic plants to assess potential effects of *PpMYBS3* on below-ground development (**Figure 8**). *PpMYBS3*-overexpressing plants exhibited significantly enhanced root system development across all measured parameters: root number increased by 131.09%, root length by 118.86%, root surface area by 95.85%, and root volume by 95.74% compared to wild-type plants (all *P* < 0.05). These results indicate that *PpMYBS3* promotes root development, which may contribute to enhanced water uptake capacity under drought conditions.

### 3.10 *PpMYBS3* overexpression confers enhanced drought tolerance

**Figure 9:**
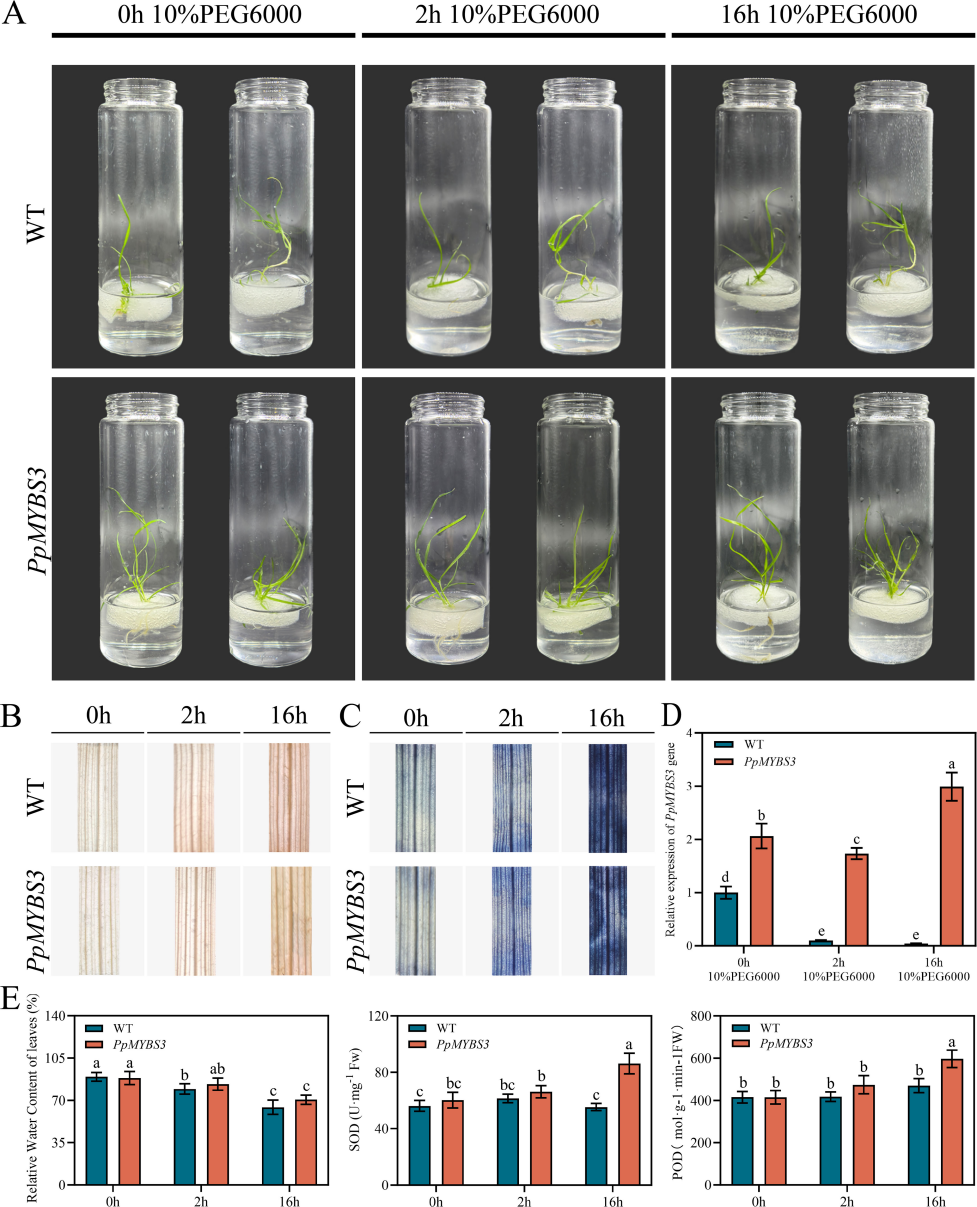
Drought tolerance analysis of transgenic Kentucky bluegrass overexpressing *PpMYBS3*. **(A)** Phenotypic changes under PEG-induced osmotic stress at 0, 2, and 16 h. **(B)** DAB staining (H_2_O_2_) at 0, 2, and 16 h. **(C)** NBT staining (O_2_⁻) at 0, 2, and 16 h. **(D)** qRT-PCR analysis of *PpMYBS3* expression dynamics under osmotic stress. **(E)** Physiological measurements: relative water content (%), SOD activity (U·g⁻¹ FW), and POD activity (U·g⁻¹ FW) in wild-type (WT) and *PpMYBS3*-overexpressing (OE) lines at 0, 2, and 16 h of PEG treatment. Data represent mean ± SD of two independent transgenic lines (OE-3 and OE-5), each with three biological replicates (total n = 6 per genotype per time point). Different lowercase letters indicate significant differences, Student’s t-test.

To directly assess the role of *PpMYBS3* in drought tolerance, wild-type and transgenic Kentucky bluegrass plants were subjected to PEG-induced osmotic stress (**Figure 9A**). Both genotypes exhibited progressive wilting with extended treatment duration; however, at 16 h of treatment, transgenic plants displayed significantly less severe wilting compared to wild-type, with leaves maintaining more upright posture.

DAB and NBT staining at multiple time points revealed markedly lower ROS accumulation in transgenic compared to wild-type plants under drought stress (**Figures 9B, C**). At 16 h of treatment, wild-type leaves exhibited intense DAB (brown) and NBT (blue) staining indicative of high H_2_O_2_ and O_2_⁻ accumulation, whereas transgenic leaves showed substantially reduced staining intensity.

qRT-PCR analysis of *PpMYBS3* expression during drought stress revealed an interesting dynamic pattern (**Figure 9D**). In transgenic plants, *PpMYBS3* expression showed an initial decrease followed by a marked increase, reaching peak levels at 16 h of treatment (2.06-fold higher than control at 0 h). This expression pattern suggests that *PpMYBS3* participates in adaptive responses to prolonged drought stress.

Physiological measurements further supported enhanced drought tolerance in transgenic plants (**Figure 9E**). As the drought stress persisted, relative water content (RWC) of both wild-type and transgenic Kentucky bluegrass leaves decreased. However, at 2 h of stress, the RWC of transgenic plants was significantly higher than that of the wild-type (*P* < 0.05). SOD and POD activities in transgenic plants were also significantly higher than those in the wild-type at 2 h and 16 h of stress (*P* < 0.05; **Figure 9E**), indicating enhanced antioxidant defense capacity. All physiological data in **Figure 9E** represent the mean ± SD of measurements from both independent transgenic lines (OE-3 and OE-5), each with three biological replicates.

## 4 Discussion

### 4.1 Wax-associated epidermal traits and drought-related responses in Kentucky bluegrass

The cuticular wax layer serves as the first line of defense against non-stomatal water loss in terrestrial plants, and its abundance has been positively correlated with drought tolerance across multiple species (Patwari et al., 2019; Zhang et al., 2019). In this study, we demonstrate that wax-associated epidermal structures vary significantly among Kentucky bluegrass cultivars and co-occur with drought tolerance performance differences. The drought-tolerant cultivar Arcadia exhibited the highest wax crystal density (38.2 ± 5.1 crystals per 100 μm^2^ on the adaxial surface), while the drought-sensitive cultivar Jenny displayed the lowest density (8.4 ± 2.6 crystals per 100 μm^2^; **Supplementary Figure 1B**). This differential wax structure pattern correlated with physiological responses under drought: Arcadia maintained higher RWC (65.3 vs. 41.4%), accumulated less ROS, exhibited lower MDA content, and showed smaller percentage declines in chlorophyll, SOD, and POD compared to Jenny (**Figure 3**; **Supplementary Table 1**).

The decline in SOD and POD activities observed at Day 20 across all cultivars merits discussion. While numerous short-term drought studies report transient increases in antioxidant enzyme activities as part of the active defense response, a decline during prolonged and severe stress is well documented and reflects the biphasic response model of antioxidant defenses (Xu et al., 2011; Blum, 2017). Under sustained severe water deficit (20 d complete withholding in our study), cumulative cellular damage, oxidative inactivation of enzyme proteins, and substrate depletion lead to net declines in measurable enzyme activity. This pattern has been specifically described in Kentucky bluegrass, where SOD activity peaked at approximately 8–12 d of drought and then declined with extended stress duration (Xu et al., 2011). Critically, the relative magnitude of these declines differed among cultivars, with Arcadia showing the smallest percentage reductions, consistent with better maintenance of antioxidant capacity in the more tolerant cultivar (**Supplementary Table 1**).

These findings are consistent with previous reports in related species. In rice, dysfunction of wax biosynthesis genes reduces cuticular wax density and compromises drought tolerance (Jian et al., 2022). In *Poa annua*, epidermal wax coverage negatively correlates with electrolyte leakage and MDA content under stress (Chalise and Merewitz, 2024). Our results extend these observations to Kentucky bluegrass and provide the first quantitative characterization of inter-cultivar variation in wax-associated epidermal structures and their association with drought tolerance in this economically important turfgrass species. Nonetheless, we emphasize that the present data establish a correlative association between wax crystal density and drought performance; direct measurement of total wax load per leaf area (e.g., by GC-MS or gravimetric analysis) and cuticular transpiration assays will be required to confirm a mechanistic link.

### 4.2 Integration of hormone signaling networks in drought response

Transcriptomic analysis revealed substantial differential expression of genes involved in auxin, ABA, and SA signaling pathways among varieties (**Figure 5**). The drought-tolerant variety Arcadia exhibited elevated expression of specific auxin signaling components, including *IAA4* and *IAA19*. These genes encode Aux/IAA proteins that function as key regulators of auxin-responsive gene expression and have been implicated in drought tolerance in other species. For example, overexpression of *OsIAA20* in rice enhances drought resistance through upregulation of ABA-responsive genes (Zhang et al., 2022). Similarly, *MdIAA9* overexpression improves osmotic stress tolerance in tobacco (Huang et al., 2019).

In the ABA signaling pathway, PP2C family genes exhibited higher expression in the drought-tolerant varieties (Arcadia and K.B.G) compared to the sensitive variety Jenny. PP2C phosphatases function as key negative regulators of ABA signaling by dephosphorylating SnRK2 kinases, thereby modulating downstream ABA-responsive gene expression (Li et al., 2024). The observed coordinated expression of PP2C and SnRK2 family members (**Figure 5D**) suggests that drought-tolerant genotypes may maintain a more responsive yet buffered ABA signaling state, enabling rapid stomatal regulation while limiting excessive stress-associated growth penalties.

In parallel, the differential expression of SA signaling components (*NPR1*/*NPR3* and *TGA* factors) indicates that hormone network reconfiguration accompanies drought adaptation (Huang et al., 2026; Kumar, 2014; Zhang et al., 2025). Although SA signaling is classically associated with pathogen defense, it has increasingly been recognized as a modulator of abiotic stress responses through redox homeostasis and transcriptional reprogramming (Zhang et al., 2018; Saleem et al., 2021). In this study, *NPR3* expression was higher in Arcadia, whereas *NPR1* showed higher expression in K.B.G (**Figure 5C**). Given the interplay between SA signaling and cellular redox status, these patterns are consistent with the reduced ROS accumulation observed in Arcadia under drought (**Figures 3B, C**), but the causal relationships require targeted functional validation in Kentucky bluegrass.

Overall, our transcriptome data support a model in which drought-tolerant varieties exhibit coordinated modulation of ABA- and auxin-related modules alongside stress-associated transcriptional networks. Importantly, rather than treating hormone signaling as an isolated finding, we interpret it here as an upstream regulatory layer that may converge on downstream structural and physiological traits, including cuticular wax deposition and stomatal behavior, that directly influence plant water status.

### 4.3 *PpMYBS3* as a candidate transcriptional regulator linking wax deposition to drought tolerance

Transcription factors are crucial components of the regulatory signaling network in plants and play a significant role in abiotic stress responses. Recent studies increasingly demonstrate that MYB proteins mediate stress signaling pathways; for instance, *AtMYB41*, *AtMYB74*, *AtMYB102*, and *AtMYB108* are pivotal for drought tolerance (Xu et al., 2015). In woody plants, *PtoMYB142* was identified as a drought-induced MYB transcription factor that directly binds to the promoters of wax biosynthesis genes *CER4* and *KCS6*, thereby promoting wax accumulation and enhancing drought resistance in poplar (Song et al., 2022).

A central objective of this study was to identify transcriptional regulators that could explain the wax-density gradient and drought tolerance differences among cultivars. Rather than employing a single criterion, we applied a multi-step filtering strategy (Section 3.7) that integrated (i) Pfam domain annotation to define the MYB family, (ii) *a priori* focus on the 1R-MYB subfamily based on published functional precedent, (iii) expression-phenotype concordance with the cultivar wax-density gradient, (iv) stress inducibility by qRT-PCR, and (v) phylogenetic proximity to functionally characterized orthologs (*OsMYBS3*, *SiMYBS3*). Among 87 MYB-family DEGs, *PpMYBS3* emerged as the strongest candidate based on the convergence of all five criteria.

Functional validation provides direct support for a positive role of *PpMYBS3* in drought adaptation. *PpMYBS3*-overexpressing Kentucky bluegrass lines (two independent lines, OE-3 and OE-5) displayed increased wax-associated epidermal structures (**Figure 7C**), enlarged papillae volume (**Figure 7E**), and reduced stomatal aperture area (**Figures 7F, G**). These structural alterations plausibly contribute to reduced transpirational water loss through two complementary routes: strengthened non-stomatal barrier function via enhanced wax structures and altered stomatal conductance via reduced aperture. Complementary qRT-PCR analysis of downstream wax biosynthesis genes revealed that *CER1*, *KCS6*, and *ABCG11* were significantly upregulated in *PpMYBS3*-overexpressing lines (**Supplementary Figure 2**), providing molecular evidence consistent with *PpMYBS3* acting as a positive transcriptional regulator of the wax biosynthetic pathway. Similar regulatory patterns have been reported for *OsMYB60* in rice (Jian et al., 2022) and *LmMYB1* in *Lolium multiflorum* (Liu et al., 2023a), suggesting that the role of MYB transcription factors in promoting wax synthesis and enhancing drought resistance may be conserved across grass species.

Notably, the subcellular localization of *PpMYBS3* to the nucleus (with detectable cytoplasmic signal; **Figure 7B**) is consistent with its putative role as a transcription factor. However, subcellular localization alone does not establish direct transcriptional targets. Therefore, the key conclusion supported by the present data is that *PpMYBS3* is sufficient to promote wax-associated epidermal traits and improve drought tolerance-related phenotypes when overexpressed, rather than establishing a fully mapped regulatory pathway. The aforementioned study on *PtoMYB142*, which demonstrated direct binding to *CER4* and *KCS6* promoters (Song et al., 2022), provides a model for future investigations to identify the direct downstream targets of *PpMYBS3* and elucidate its complete regulatory mechanism in wax synthesis and drought resistance. Specifically, chromatin immunoprecipitation (ChIP-qPCR), electrophoretic mobility shift assays (EMSA), and dual-luciferase reporter assays should be employed to test whether *PpMYBS3* directly binds to the promoters of *CER1*, *KCS6*, and *ABCG11*, and whether it acts by suppressing the wax repressor DEWAX, as reported for the *Arabidopsis* MYB-SHAQKYF family members *MYS1* and *MYS2* (Liu et al., 2022).

### 4.4 *PpMYBS3* improves drought performance via coordinated above- and below-ground traits

Drought tolerance is a systems-level trait integrating water acquisition, retention, and stress damage mitigation (Harfouche et al., 2014). Beyond leaf surface modifications, *PpMYBS3* overexpression significantly enhanced root system architecture, including root number (+131.09%), length (+118.86%), surface area (+95.85%), and volume (+95.74%; **Figure 8**). Increased root exploration capacity can improve water uptake under soil drying conditions and is a common feature of drought-adapted genotypes. Similar findings have been reported for *GmMYB84* in soybean, which enhances drought resistance by regulating primary root elongation (Wang et al., 2017), and *LcMYB2* from *Leymus chinensis*, which promotes root growth under drought stress (Zhao et al., 2019).

Additionally, transgenic plants accumulated less ROS under PEG-induced stress, as indicated by reduced DAB and NBT staining intensities (**Figures 9B, C**). Although these assays are semi-quantitative, the consistently lower staining in transgenic lines across time points supports reduced oxidative burden. Quantitative physiological measurements corroborated these observations: transgenic plants maintained significantly higher RWC and SOD/POD activities than wild-type plants at both 2 h and 16 h of PEG treatment (**Figure 9E**). Together with the cultivar comparison results (**Figure 3**), these findings suggest that wax-associated epidermal protection and improved water relations may indirectly reduce stress-induced ROS accumulation, while enhanced antioxidant capacity could further limit oxidative damage. Future work should quantify ROS and lipid peroxidation using biochemical assays (e.g., spectrophotometric H_2_O_2_ content, O_2_^-^ production rate, and MDA quantification) to strengthen this mechanistic layer.

Importantly, PEG-induced osmotic stress is an established proxy for drought stress but does not fully replicate soil drying dynamics. Nonetheless, the convergence of improved morphology (wax-associated structures and stomatal traits), upregulation of wax biosynthesis genes, improved root architecture, enhanced antioxidant enzyme activities, higher relative water content, and reduced ROS accumulation provides a coherent phenotype package supporting the role of *PpMYBS3* in drought adaptation.

## 5 Conclusions

This study links natural variation in leaf cuticular wax-associated structures to drought performance in Kentucky bluegrass and, through comparative transcriptomics combined with a multi-criteria candidate gene selection strategy, identifies *PpMYBS3* as a drought-associated 1R-MYB candidate. Stable overexpression of *PpMYBS3* in two independent transgenic lines enhances wax-related epidermal traits, upregulates canonical wax biosynthesis genes (*CER1*, *KCS6*, *ABCG11*), reduces stomatal aperture, promotes root system development, maintains higher relative water content, and mitigates ROS accumulation under osmotic stress, collectively improving drought-associated phenotypes. *PpMYBS3* therefore represents a promising genetic resource for drought-resilient turfgrass improvement and a foundation for future mechanistic dissection - including direct wax quantification (GC-MS profiling), cuticular transpiration assays, and ChIP-based identification of direct transcriptional targets - of the transcriptional control of epidermal barrier formation and root architecture under water deficit.

## Data Availability

The datasets generated and analyzed during the current study are available in the **Supplementary Materials**. The transcriptome data were deposited in the NCBI Sequence Read Archive (SRA) under accession PRJNA1217998.
