# Supplementary material for: Integrative transcriptomics nominates a 1R-MYB candidate associated with epidermal wax-related traits and osmotic-stress responses in Kentucky bluegrass
Source: Front Plant Sci. 2026 May 20;17:1817480. doi: 10.3389/fpls.2026.1817480 (PMC13229787; doi:10.3389/fpls.2026.1817480)
Supplement: Supplementary file 7 [file Supplementaryfile1.docx]

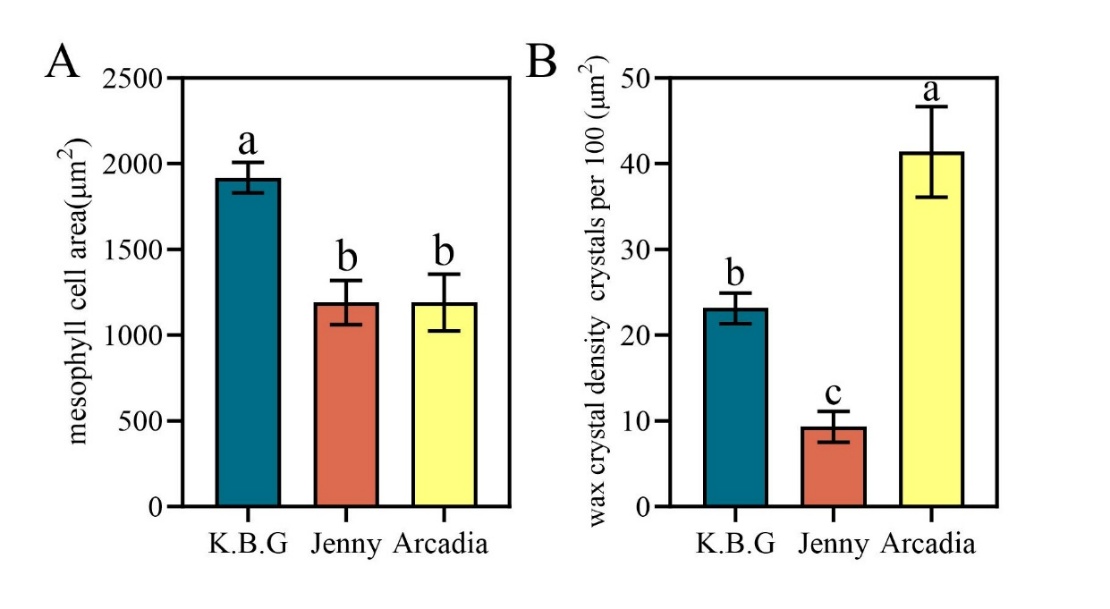


**Figure S1** Mesophyll cell area and leaf epidermal wax density in three Kentucky bluegrass cultivars. Values represent mean ± SD (n = 3). Different letters indicate significant differences at *P* < 0.05 (Tukey’s HSD).


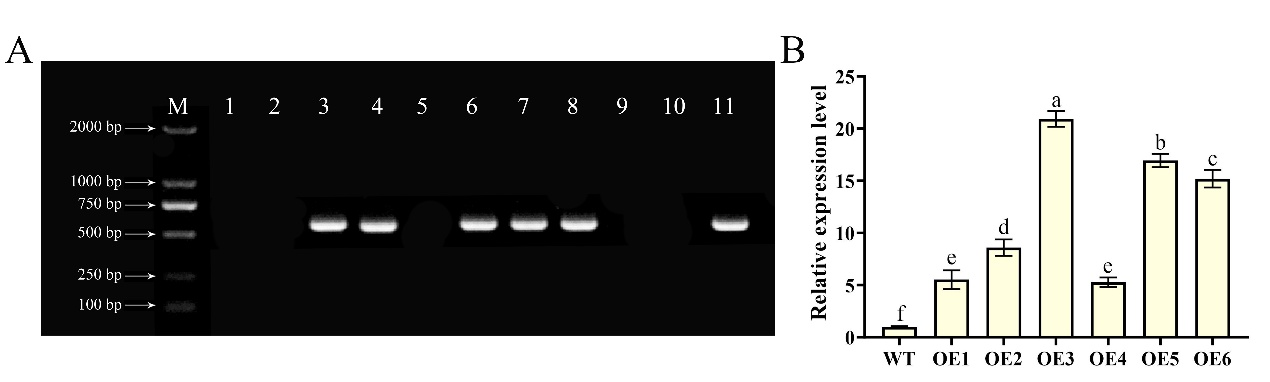


**Figure S2** Acquisition of overexpressed *PpMYBS3* Kentucky bluegrass plant. A: DNA molecular detection of overexpression lines (M: DNA Maker DL 2000; 1: Blank control; 2: WT; 3-11: *PpMYBS3* gene overexpression plants OE1-OE6.); B: Expression analysis of *PpMYBS3* in overexpressed transgenic Kentucky bluegrass. Different lowercase letters indicate significant differences, Student’s t-test.


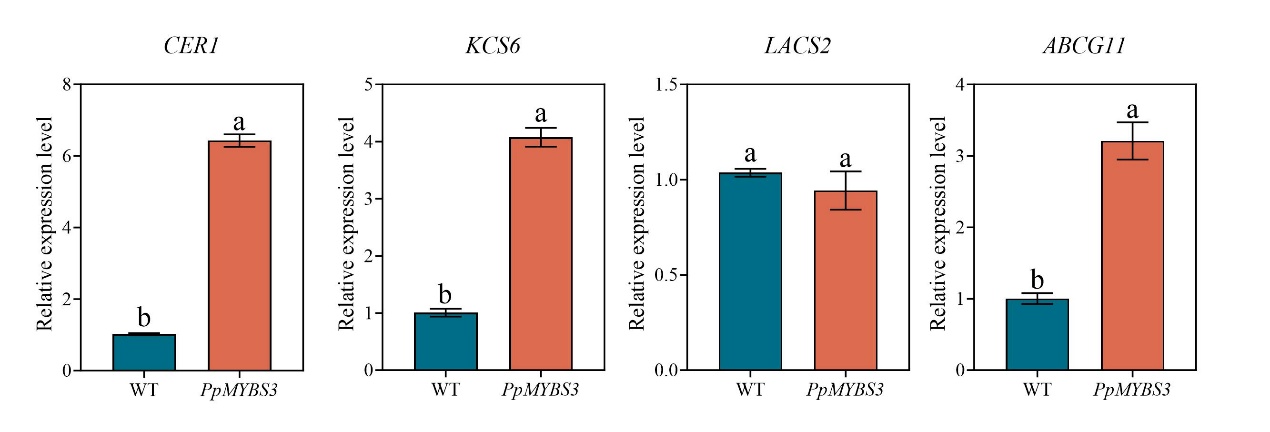


**Figure S3** Wax synthesis and transport gene expression analysis. Different lowercase letters indicate significant differences, Student’s t-test.


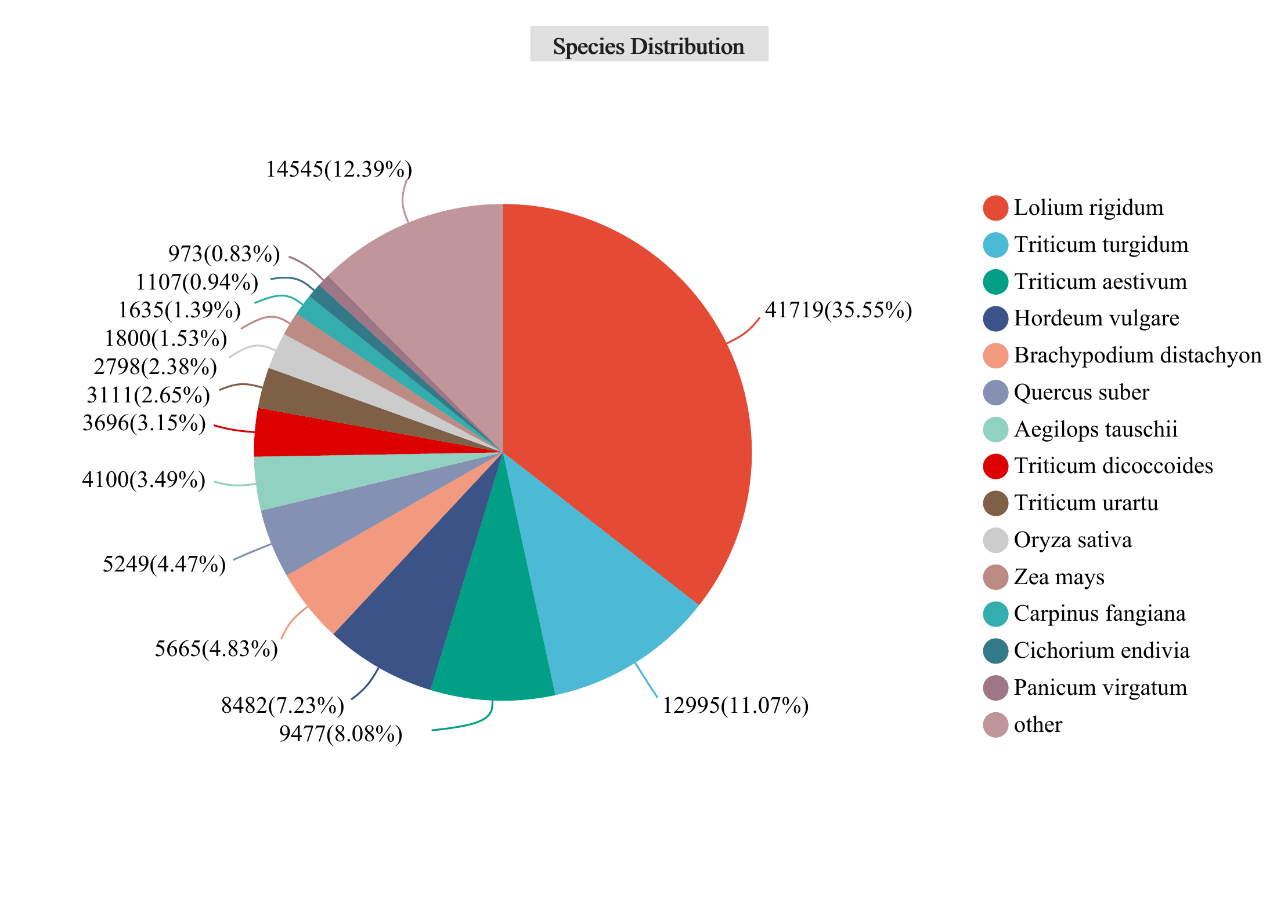


**Figure S4** NR notes species distribution


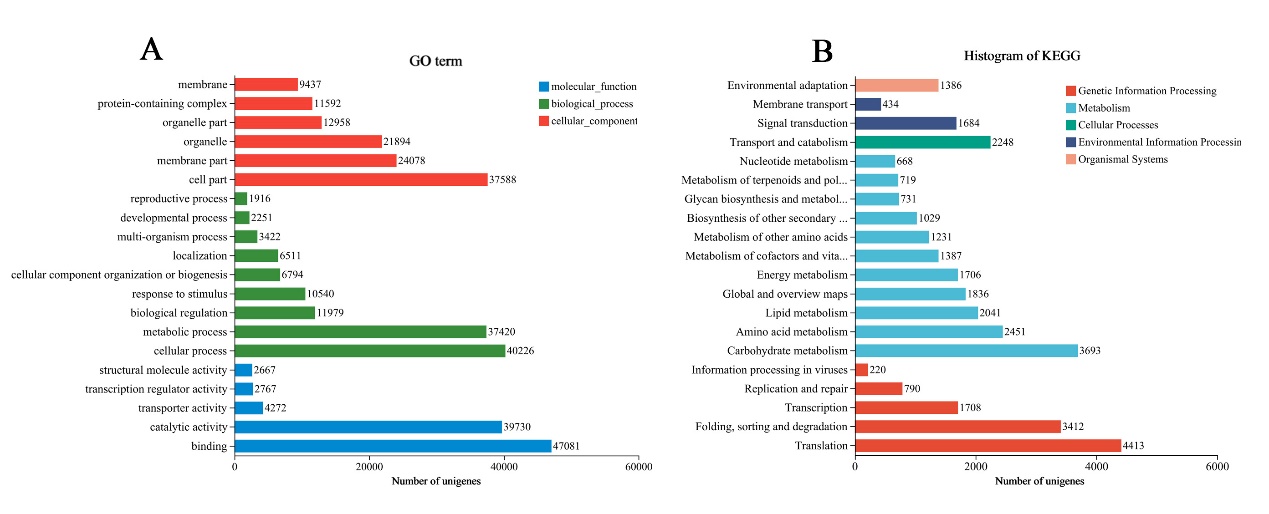


**Figure S5** (A) Bar plots of Gene Ontology (GO) classifications of the assembled sequences. (B) Plots showing categories of genes classified based on the Kyoto Encyclopedia of Genes and Genomes (KEGG) analysis.


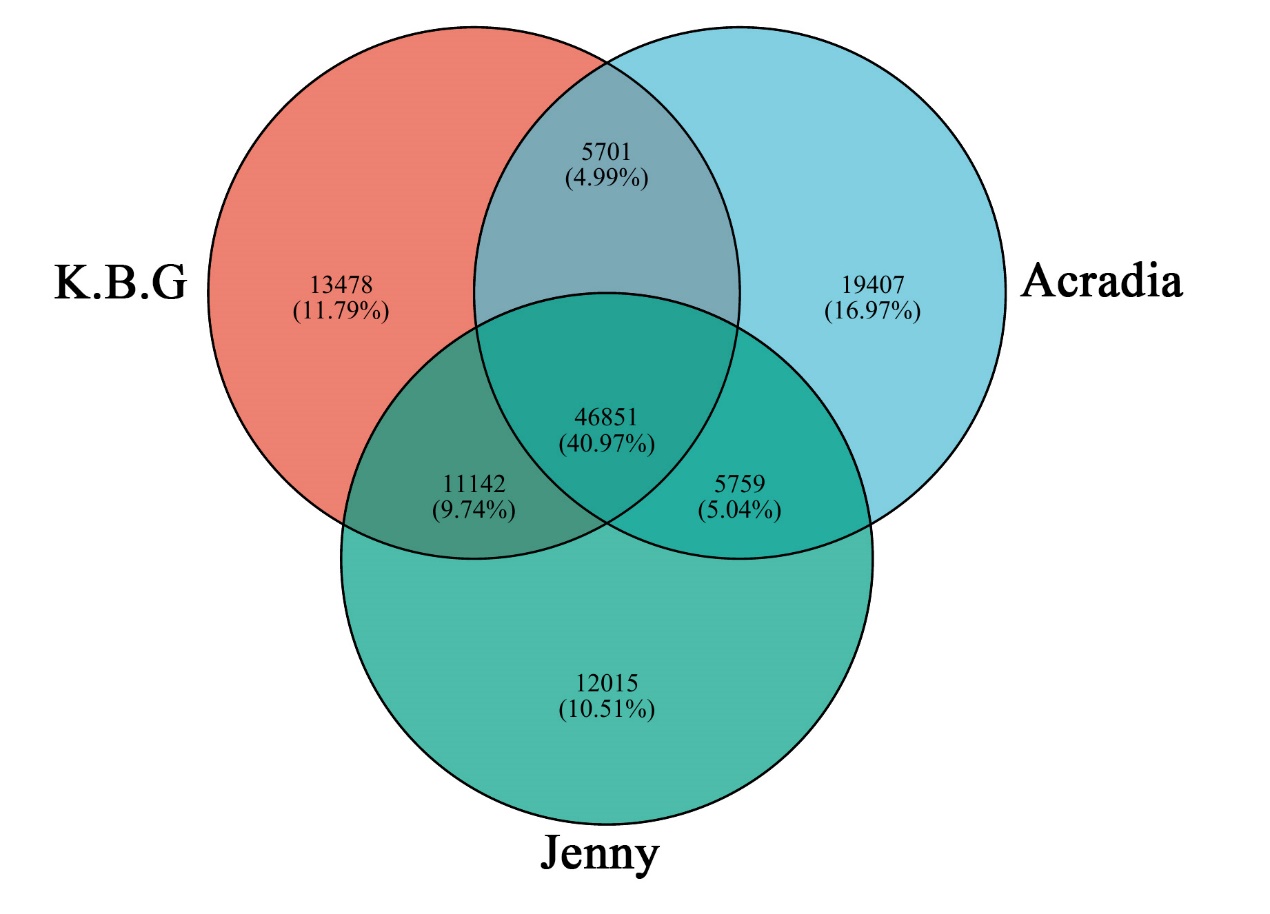


**Figure S6** Venn analysis of expression levels between samples.


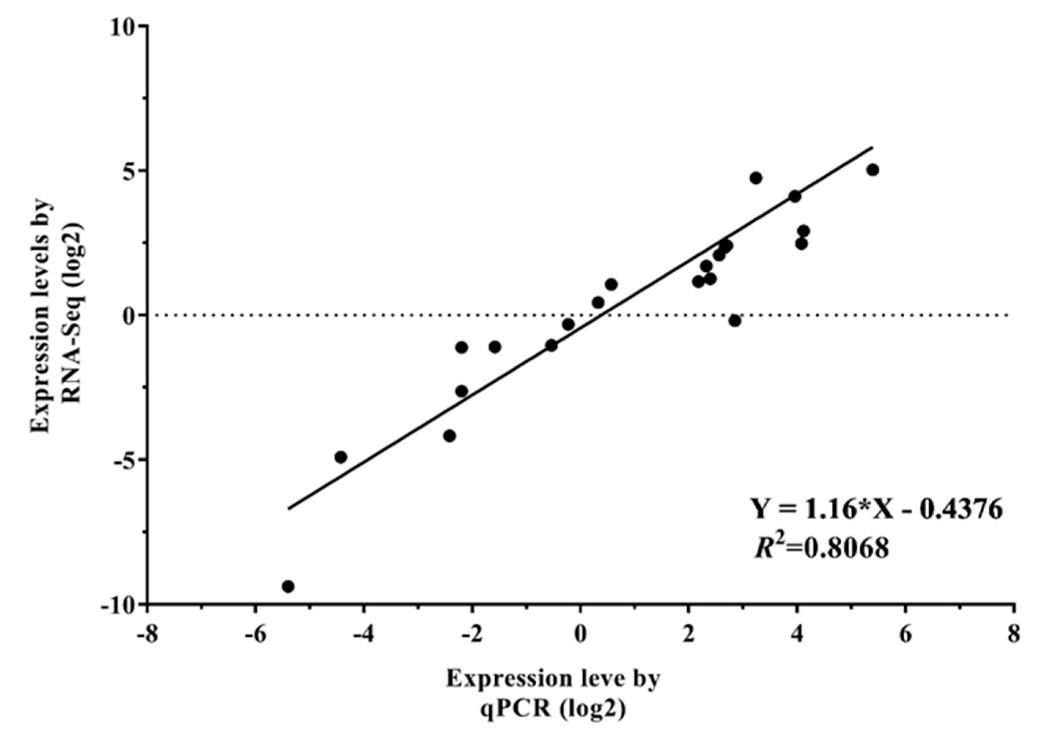


**Figure S7** Transcriptome data verification.
